# Transcription factor 7 like 2 promotes metastasis in hepatocellular carcinoma via NEDD9-mediated activation of AKT/mTOR signaling pathway

**DOI:** 10.1186/s10020-024-00878-9

**Published:** 2024-07-25

**Authors:** Linsong Tang, Shengjun Xu, Rongli Wei, Guanghan Fan, Junbin Zhou, Xuyong Wei, Xiao Xu

**Affiliations:** 1 Department of Urology, The Second Affiliated Hospital, Zhejiang University School of Medicine, Hangzhou, Zhejiang China; 2 Institute of Translational Medicine, Zhejiang University, Hangzhou, Zhejiang China; 3 Department of Hepatobiliary & Pancreatic Surgery and Minimally Invasive Surgery, Zhejiang Provincial People’s Hospital (Affiliated People’s Hospital), School of Clinical Medicine, Hangzhou Medical College, Hangzhou, Zhejiang China; 4 Department of Hepatobiliary and Pancreatic Surgery, Affiliated Hangzhou First People’s Hospital, School of Medicine, Westlake University, Hangzhou, Zhejiang China; 5 Department of Hepatobiliary and Pancreatic Surgery, Lishui People’s Hospital, Lishui, Zhejiang China; 6 NHC Key Laboratory of Combined Multi-Organ Transplantation, Hangzhou, Zhejiang China

**Keywords:** Hepatocellular carcinoma, TCF7L2, NEDD9, AKT/mTOR pathway, Tumor metastasis

## Abstract

**Background:**

Hepatocellular carcinoma (HCC) is one of the most common malignant tumors of the digestive system, and the exact mechanism of HCC is still unclear. Transcription factor 7 like 2 (TCF7L2) plays a pivotal role in cell proliferation and stemness maintenance. However, the exact mechanism of TCF7L2 in HCC remains unclear.

**Methods:**

Clinical samples and public databases were used to analyze the expression and prognosis of TCF7L2 in HCC. The function of TCF7L2 in HCC was studied in vitro and in vivo. ChIP and luciferase assays were used to explore the molecular mechanism of TCF7L2. The relationship between TCF7L2 and NEDD9 was verified in HCC clinical samples by tissue microarrays.

**Results:**

The expression of TCF7L2 was upregulated in HCC, and high expression of TCF7L2 was associated with poor prognosis of HCC patients. Overexpression of TCF7L2 promoted the metastasis of HCC in vitro and in vivo, while Knockdown of TCF7L2 showed the opposite effect. Mechanically, TCF7L2 activated neural precursor cell expressed developmentally downregulated protein 9 (NEDD9) transcription by binding to the -1522/-1509 site of the NEDD9 promoter region, thereby increasing the phosphorylation levels of AKT and mTOR. The combination of TCF7L2 and NEDD9 could distinguish the survival of HCC patients.

**Conclusions:**

This study demonstrated that TCF7L2 promotes HCC metastasis by activating AKT/mTOR pathway in a NEDD9-dependent manner, suggesting that potential of TCF7L2 and NEDD9 as prognostic markers and therapeutic targets for HCC.

**Supplementary Information:**

The online version contains supplementary material available at 10.1186/s10020-024-00878-9.

## Background

HCC accounts for ~ 80% of liver cancer and represents the third cancer-related mortality globally (Sung et al. 2021). Surgery is still the most important treatment for early-stage HCC patients, with a five-year survival rate exceeding 70% (Tang et al. 2022). Multidisciplinary comprehensive treatment including radiotherapy, targeted therapy and immunotherapy is currently the main therapeutic strategy of advanced-stage HCC patients (Tang et al. 2020). Although great progress has been made in the treatment of HCC, some advanced-stage HCC patients do not benefit significantly from the current treatment options (Zhang et al. 2022a, b). Therefore, further elucidation of the mechanisms of HCC is required.

The Wnt/β-catenin pathway is one of the most frequently mutated pathways in HCC, with about half of HCC showing activation of the Wnt/β-catenin pathway (Vilchez et al. 2016). After Wnt activation, β-catenin enters the nucleus and binds to TCF/LEF family proteins to activate the transcription of downstream target genes, which plays an important role in the progression and metastasis of HCC (Anastas and Moon 2013). TCF7L2, also known as transcription factor 4 (TCF4), plays a crucial role in embryonic development, cell stemness regulation and tissue renewal (Hrckulak et al. 2016). TCF7L2 contains a β-catenin binding domain that functions as a downstream effector of the Wnt pathway (Jin 2016). Previous studies have linked TCF7L2 to diseases such as diabetes, obesity, and metabolic syndrome (Zhang et al. 2021). Indeed, TCF7L2 is considered to be the strongest risk factor for type 2 diabetes (Lee et al. 2023). Recent studies have also shown that TCF7L2 can affect the progression of cancer. For example, functional deficiency of TCF7L2 promotes metastasis of colorectal cancer (Wenzel et al. 2020). In gastric cancer, TCF7L2 promotes the proliferation and metastasis of cancer cell by activating downstream target genes (Zhang et al. 2022a, b). TCF7L2 has also been reported to be involved in the tumorgenesis of prostate cancer and breast cancer (Wang et al. 2021; Drake et al. 2014). It has been found that the tumorigenic ability of HCC cell lines is decreased after knocking down the expression of TCF7L2 (Malakar et al. 2019). However, the specific mechanism of TCF7L2 in HCC remains unclear.

In present study, we found that the expression of TCF7L2 was upregulated in HCC. Knockdown of TCF7L2 could significantly inhibit the invasion and metastasis of HCC. Furthermore, we clarified that TCF7L2 promotes the progression of HCC via NEDD9-dependent regulation of AKT/mTOR pathway. The combination of TCF7L2 and NEDD9 could distinguish the survival of HCC patients.

## Materials and methods

### Data collection pre-processing

The mRNA expression data of TCF7L2 in GSE14520, GSE46465 were afforded from GEO (Gene expression omnibus) database and processed by GEO2R. The expression and clinical information of HCC patients were obtained from TCGA (The Cancer Genome Atlas) cohort (https://portal.gdc.cancer.gov/). The data of the interaction between transcription factors and target genes were obtained from three databases: hTFtarget (http://bioinfo.life.hust.edu.cn/hTFtarget/ ), CHEA (https://maayanlab.cloud/Harmonizome/) and ChIPBase (http://rna.sysu.edu.cn/chipbase/), respectively(Zhang et al. 2020; Lachmann et al. 2010; Rouillard et al. 2016; Zhou et al. 2017a, b).

### Clinical samples

The cancer tissues and normal liver tissues in this study were obtained from HCC patients who underwent surgical resection in the First Affiliated Hospital, Zhejiang University School of Medicine. Patients were included with criteria: pathological diagnosis of HCC; did not receive preoperative radiotherapy, chemotherapy and other treatment; adjacent normal liver tissues were isolated from at least 2 cm away from the cutting edge; had complete postoperative follow-up data. Samples from a total of 87 HCC patients were fabricated into tissue microarray for subsequent expression and survival analysis. The clinical characteristics of the 87 patients were shown in Table S1. The patients involved in this study had signed an informed consent form. This study was approved by the Ethics Committee of the First Affiliated Hospital, Zhejiang University School of Medicine.

### Cell culture

Human cell lines of HCC, including Hep3B, Li-7, HepG2, Huh7, PLC/PRF/5 and SK-Hep-1 were purchased from Shanghai Cell Bank, Chinese Academy of Sciences. HCCLM3, MHCC97-H, MHCC97/L and SNU-449 were purchased from American Type Culture Collection, USA. HCC cell lines were cultured in appropriate medium supplemented with 10% fetal bovine serum (FBS) at 37 °C in humidified air containing 5% CO_2_.

### Western blotting

Total cell protein was extracted from HCC cells and lysed in lysis buffer. The protein samples were separated by SDS-PAGE (Sodium Dodecyl Sulfate-Polyacrylamide Gel Electrophoresis), transferred to PVDF (Polyvinylidene Fluoride) membranes, and blocked in TBST containing 5% skim milk. Then the membranes were probed with primary antibodies and secondary antibodies separately. The membranes were detected by Image Lab software (Bio-Rad, Hercules, CA). In total, primary antibodies included: β-actin (66009-1-Ig, Proteintech), TCF7L2(#2565, Cell Signaling Technology), NEDD9(#4044, Cell Signaling Technology), phospho-AKT (66444-1-Ig, Proteintech), total-AKT (60203-2-Ig, Proteintech), phospho-mTOR (67778-1-Ig, Proteintech), total-mTOR (66888-1-Ig, Proteintech).

### Quantitative real-time PCR

Total RNA of HCC cells was extracted by Cell/Tissue Total RNA Kit (19221ES50, Yeasen), and quantitative real-time PCR was performed. All operations followed the manufacturer’s protocol. Primer sequences (Tsingke Biological Technology) used in this study were listed in Table S2.

### Immunohistochemical (IHC) staining

The immunohistochemical staining was performed as the previously descripted (Zhuo et al. 2021). The primary antibodies against TCF7L2(#2565, Cell Signaling Technology), NEDD9(AP14592c, Abcepta) were used at concentrations of 1:500 and 1:100, respectively. IHC score was calculated by cell staining intensity multiplied the proportion of positive cells. Cell staining intensity was defined as follows: 0 (none), 1 (pale yellow), 2 (brownish yellow) and 3 (brown). Proportion of positive cells was defined as: 1 (0–25%), 2 (26–50%), 3 (51–75%) and 4 (> 75%). IHC score less than 7 (≤ 7) was defined as low expression, whereas the others represented high expression. All stained slides were determined independently by two pathologists.

### Gene overexpression or knockdown

TCF7L2 overexpression lentivirus was produced by OBiO Technology. Small hairpin RNA (shRNA) targeting TCF7L2 knockdown lentivirus was produced by GeneChem. Small interfering RNA (siRNA) targeting NEDD9 was designed by GenePharma. For stable cell clone, after the growth of lentivirus-transfected cells reached 80–90%, puromycin was used for screening. siRNA was transfected by jetPRIME^®^ (Polyplus) following the manufacturer’s protocol. The sequences were listed in Table S2.

### Cell growth and metastasis assays

Cell proliferation was assessed by Cell counting kit-8 (CCK8) assay (Yeasen). Cell metastasis was assessed by wound healing assay, transwell migration and invasion assays using 24-well Transwell chamber system (Corning). The experimental method was descripted in the previous study (Chen et al. 2021).

### ChIP-qPCR

SimpleChIP^®^ Enzymatic Chromatin IP Kit (Cell Signaling Technology) was used for Chromatin Immunoprecipitation (ChIP). The chromatin complexes were co-immunoprecipitated by anti-TCF7L2 or IgG antibodies, and the bound DNA fragments were analyzed by qPCR. Primer sequences of human NEDD9 promoter were provided in Table S2.

### Luciferase reporter assays

A 2000 bp of NEDD9 promoter fragment was cloned into the pGL4.21 Vector (NEDD9-WT), and a NEDD9 promoter fragment with random mutation of binding site was constructed into pGL4.21 Vector (NEDD9-MUT), both of them were synthesized and sequenced by Synbio Biotechnology Co., LTD. The two plasmids and pRL-TK Vector (with renilla luciferase) were transfected into cells, respectively. After 24 h, cells were lysed and then assayed for luciferase activities with the Dual-Glo^®^Luciferase Assay System (Promega). The relative luciferase intensity of the reporter gene was the ratio of firefly luciferase to renilla luciferase.

### Animal experiments

Orthotopic HCC implantation models and lung metastasis models were performed as previous study reported (Zuo et al. 2021). All animal experiments followed the guidelines of the First Affiliated Hospital, Zhejiang University School of Medicine.

### Statistical analysis

Analysis of data were performed using SPSS 22 and GraphPad Prism 8. All data were presented as the mean ± SD. Student’s t-test was used for comparing the differences of two groups. The survival of HCC patients was evaluated by log-rank test and Kaplan-Meier analysis. Comparison of clinical information was performed by χ2 test. In present study, *P* < 0.05 was considered statistically significant.

## Results

### High expression of TCF7L2 correlates with poor prognosis of HCC patients

**Fig. 1:**
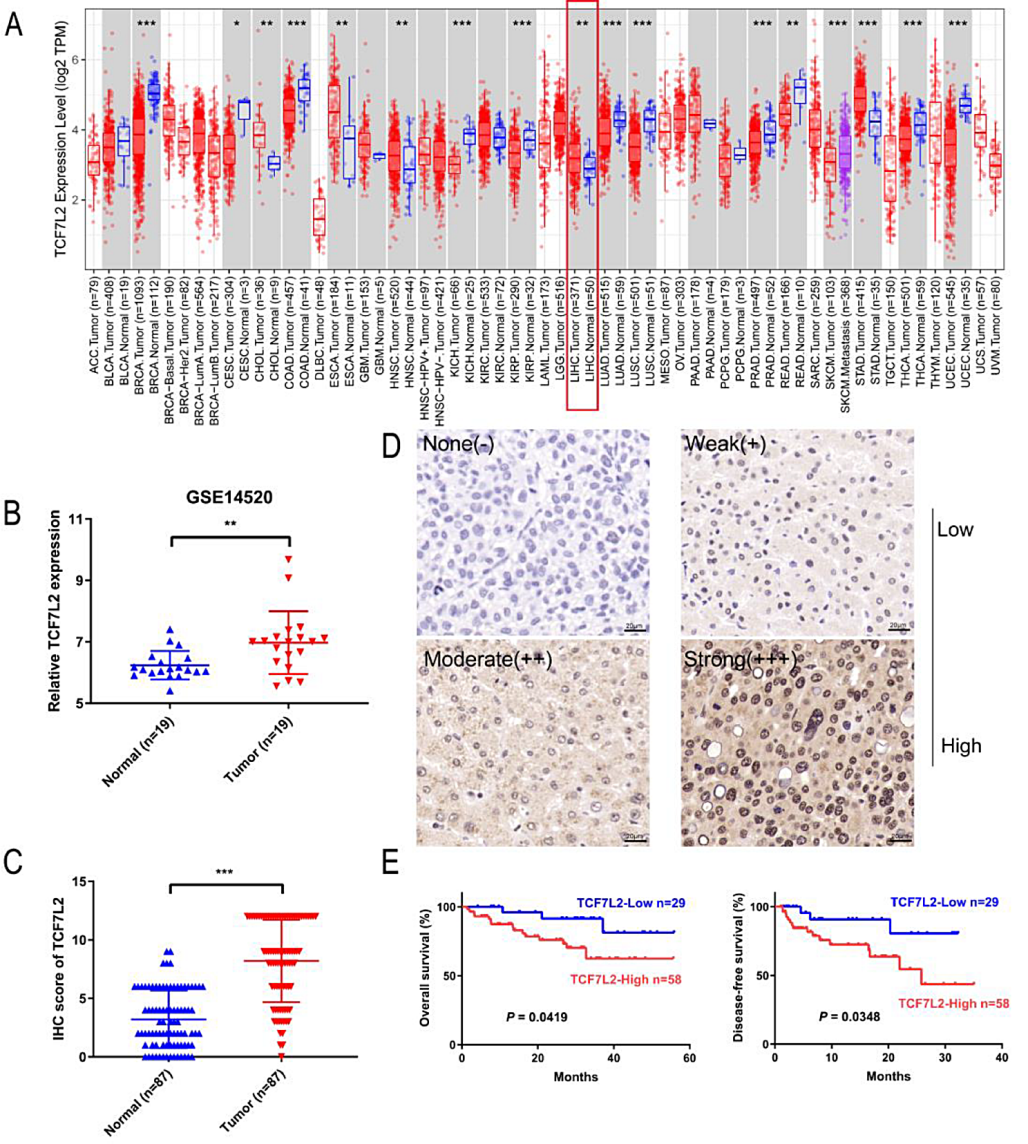
High expression of TCF7L2 correlates with poor prognosis in HCC. **A** and **B**. The mRNA expression level of TCF7L2 in different tumors tissues and normal tissues from TCGA database (**A**) and GSE14520 cohort (**B**). **C.** Immunohistochemical staining and IHC scores of TCF7L2 on the tissue microarrays of 87 HCC patients (*n* = 87). **D** and **E.** 87 HCC patients were divided into TCF7L2-low expression group and TCF7L2-high expression group according to IHC scores (**D**), and Kaplan-Meier survival analysis was performed on overall survival and disease-free survival of the two groups (**E**). * *P* < 0.05, ** *P* < 0.01, *** *P* < 0.001

The expression level of TCF7L2 in different tumors tissues and normal tissues from TCGA database was obtained by the TIMER 2.0 (Li et al. 2020). The expression of TCF7L2 was downregulated in breast cancer, colorectal cancer and prostate cancer, while it was upregulated in cholangiocarcinoma, HCC, and gastric cancer (Fig. 1A). Paired analysis of patients from the GSE14520 cohort (*n* = 19) and TCGA (*n* = 50) also yielded the same results (Fig. 1B and Figure S1A). Then, we performed immunohistochemical staining on the tissue microarrays of 87 HCC patients, which exhibited a significant upregulation of TCF7L2 in HCC tissues compared with adjacent normal liver tissues (Fig. 1C).

87 HCC patients were categorized into TCF7L2-low expression group (*n* = 29) and TCF7L2-high expression group (*n* = 58) based on immunohistochemical scores (Fig. 1D). The overall survival and disease-free survival of HCC patients with TCF7L2-high expression were significantly lower than those with TCF7L2-low expression (Fig. 1E). We also found that high TCF7L2 expression was significantly associated with vascular invasion (*P* = 0.0003) (Table 1). Taken together, these data link high TCF7L2 expression to a worse prognosis in HCC patients.

**Table 1:** nCorrelation between the expression level of TCF7L2 and clinicopathologic characteristics of 87 HCC patients

Characteristics		TCF7L2 expression		*P* value
		High(*n* = 58)	Low(*n* = 29)	
Gender	Male	54	26	0.577
	Female	4	3	
Age(years)	> 50	41	17	0.260
	≦ 50	17	12	
Tumor size(cm)	> 5	29	11	0.287
	≦ 5	29	18	
Tumor number	Single	45	22	0.857
	Multiple	13	7	
Cirrhosis	Yes	30	15	0.999
	No	28	14	
HBsAg	Positive	54	24	0.135
	Negative	4	5	
AFP(ng/mL)	> 400	27	12	0.647
	≦ 400	31	17	
Vascular invasion	Yes	40	8	0.0003***
	No	18	21	
TNM stage	I&II	31	21	0.089
	III&IV	27	8	

*Note* χ2 test were used for comparing the clinical characteristics in TCF7L2-low expression group and TCF7L2-high expression group, *** *P* < 0.001. *Abbreviations* HBsAg, hepatitis B virus surface antigen; AFP, α-Fetoprotein

### **Knockdown of TCF7L2 inhibits metastasis of HCC cells ** in vitro **and **in vivo

**Fig. 2:**
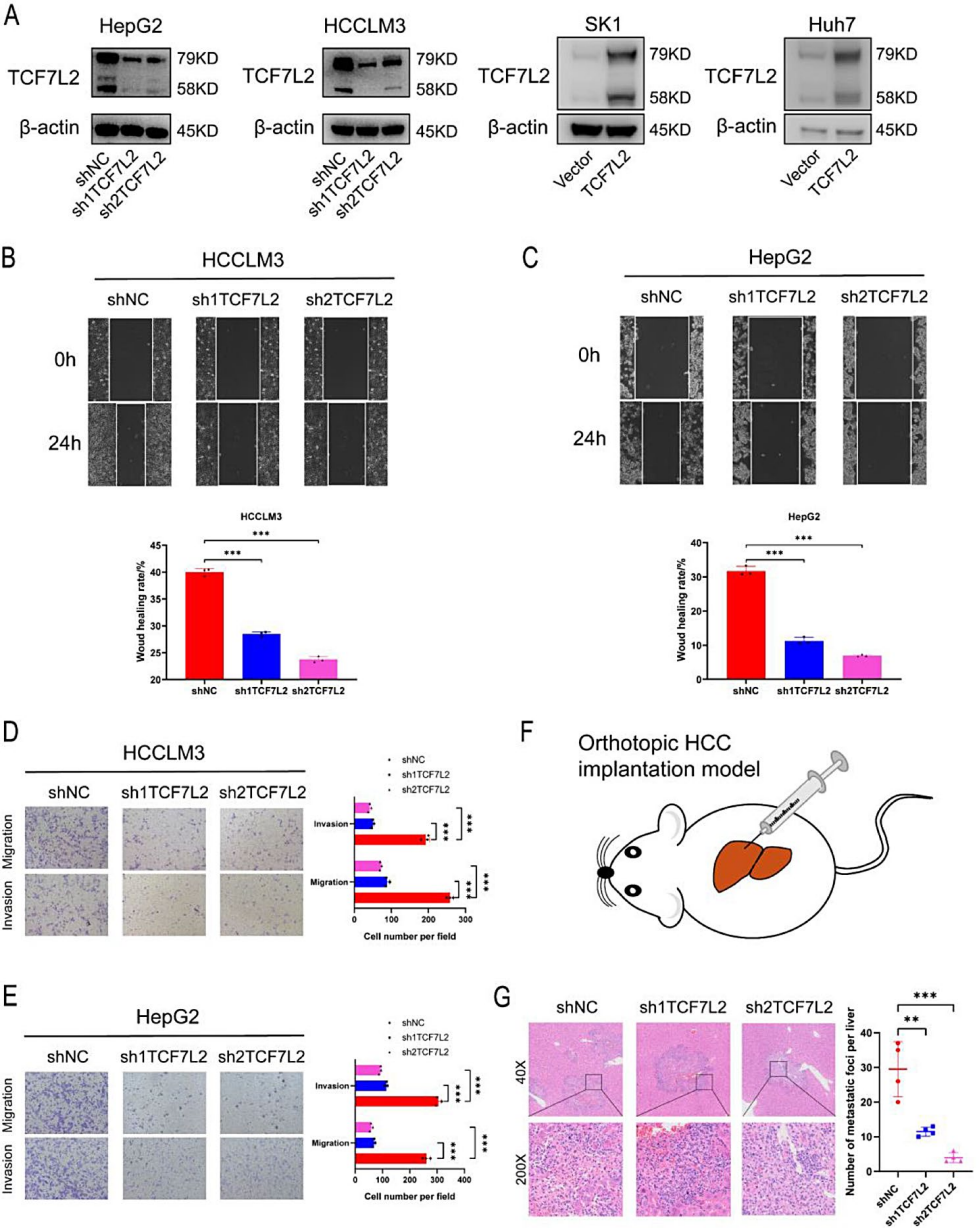
Knockdown of TCF7L2 inhibits metastasis of HCC cells in vitro and in vivo. **A**. The knockdown and overexpression effects of TCF7L2 in HCC cell lines were verified at the protein level. **B** and **C.** Knockdown of TCF7L2 inhibits migration of HCCLM3 (**A**) and HepG2 (**B**) cells detected by wound healing assay (*n* = 3 biological replicates). **D** and **E.** Knockdown of TCF7L2 attenuates the ability of HCCLM3 (**C**) and HepG2 (**D**) cells to migrate and invade detected by transwell assays (*n* = 3 biological replicates). **F.** The schematic illustration for establishing orthotopic HCC implantation models. **G.** The number of intrahepatic metastases was significantly reduced after TCF7L2 knockdown (*n* = 3 biological replicates). ** *P* < 0.01, *** *P* < 0.001

Owing to the expression of TCF7L2 varying from different HCC cell lines (Figure S1B), we selected HepG2 and HCCLM3 cell lines for TCF7L2 knockdown experiments, SK-Hep-1 (abbreviated as SK1) and Huh7 cell lines for TCF7L2 overexpression experiments. Stable cell models of TCF7L2 knockdown or overexpressed were validated at mRNA (Figure S2A) and protein levels (Fig. 2A). CCK8 assays indicated that TCF7L2 did not significantly affect the proliferation of HCC cells in vitro (Figure S2B, C). However, the wound healing assay, transwell migration and invasion assays uncovered that in HepG2 and HCCLM3 cell lines, the migration and invasion ability of cells was significantly inhibited after TCF7L2 knockdown (Fig. 2B-E). Next, HCCLM3 cells with stable TCF7L2 knockdown were injected into the liver of mice to construct orthotopic HCC implantation models (Fig. 2F), and the tumor metastasis in the liver of mice was observed 8 weeks later. We found that the number of intrahepatic metastases was significantly reduced after TCF7L2 knockdown (Fig. 2G).

### Overexpression of TCF7L2 promotes metastasis of HCC cells in vitro and in vivo

**Fig. 3:**
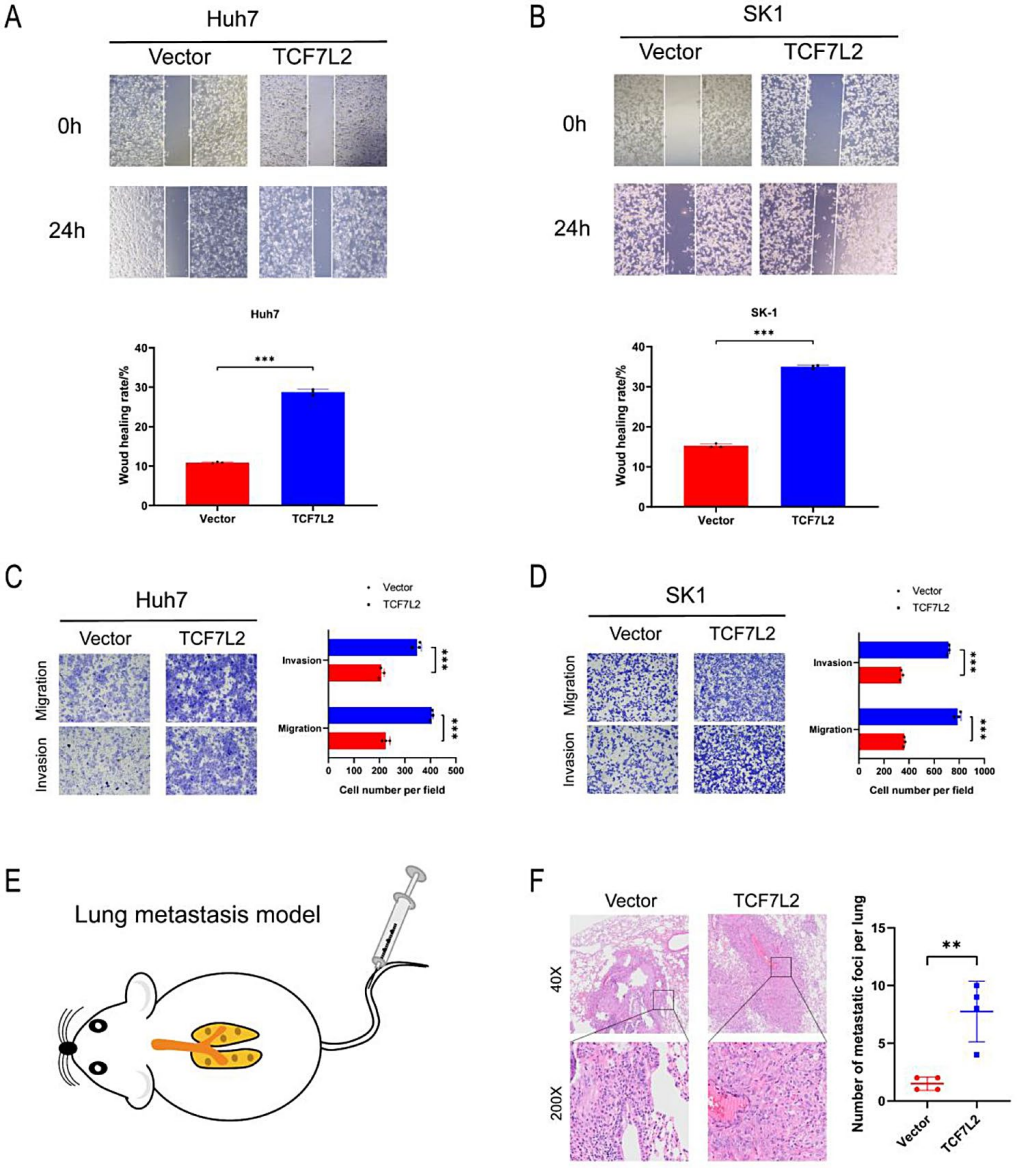
Overexpression of TCF7L2 promotes metastasis of HCC cells in vitro and in vivo. **A** and **B**. Overexpression of TCF7L2 promotes migration of Huh7 (**A**) and SK1 (**B**) cells detected by wound healing assay (*n* = 3 biological replicates). **C** and **D.** Overexpression of TCF7L2 attenuates the ability of Huh7 (**C**) and SK1 (**D**) cells to migrate and invade detected by transwell assays (*n* = 3 biological replicates). **E.** The schematic illustration for establishing lung metastasis models. **F.** The number of lung metastases was significantly increased after TCF7L2 overexpression (*n* = 4 biological replicates). ** *P* < 0.01, *** *P* < 0.001

To further validate the role of TCF7L2 in HCC, cell lines withstable TCF7L2 overexpression were used to perform wound healing assay, transwell migration and invasion assays. We found that in Huh7 and SK1 cell lines, the metastasis ability of cells was significantly enhanced after TCF7L2 overexpression (Fig. 3A-D). In addition, SK1 cells with stable TCF7L2 overexpression were injected into the tail veins of mice to construct lung metastasis models (Fig. 3E), and the tumor metastasis in the lung of mice was observed 8 weeks later. The number of lung metastases was significantly increased after TCF7L2 overexpression compared to the control group (Fig. 3F). Collectively, these data suggest that TCF7L2 promotes HCC metastasis in vivo and in vitro.

### TCF7L2 regulates HCC migration and invasion by activating AKT/mTOR pathway

**Fig. 4:**
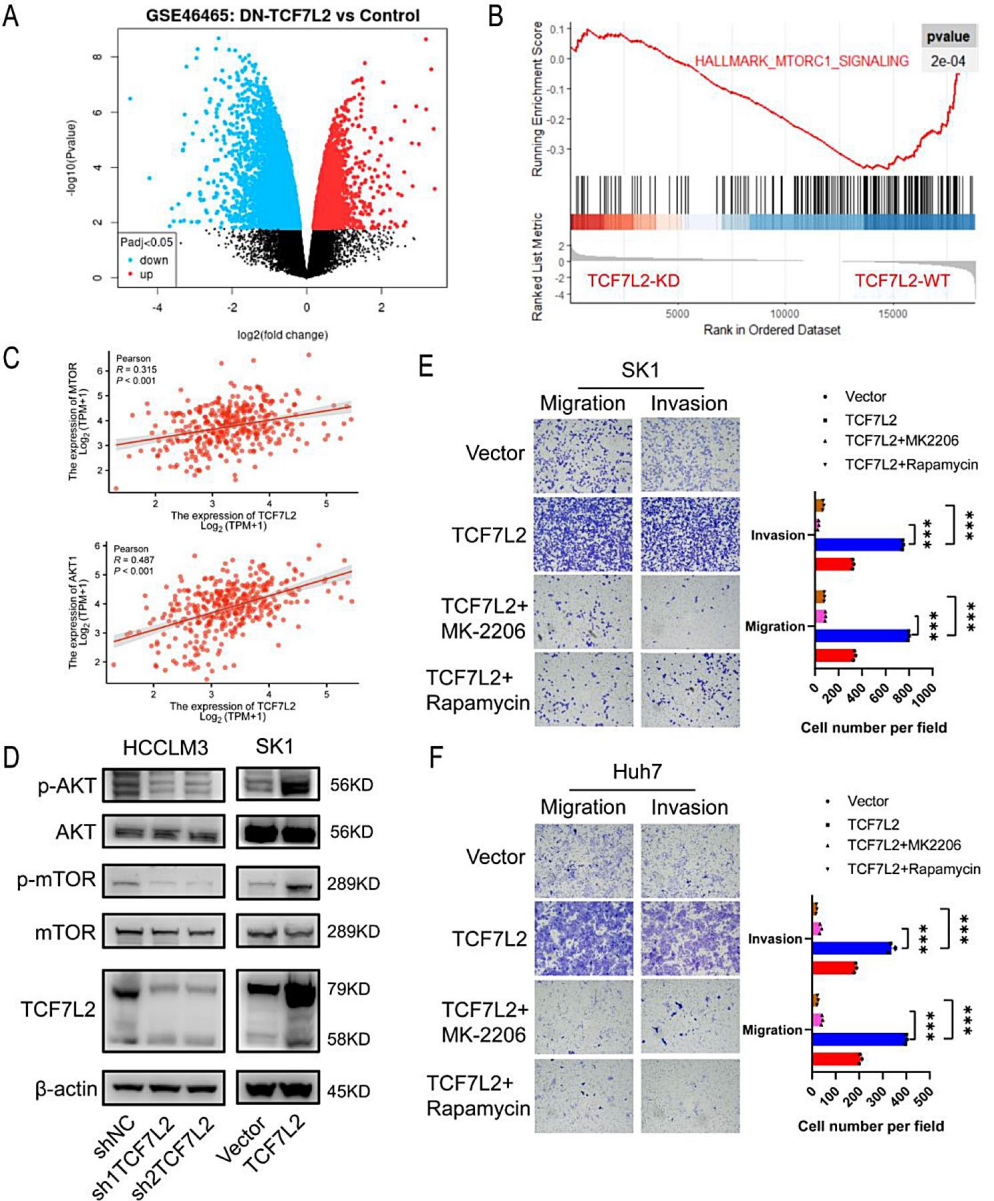
TCF7L2 promotes HCC migration and invasion by activating AKT/mTOR pathway. **(A)** Differentially expressed genes (DEGs) between the TCF7L2 knockdown group and the control group in the GSE46465 dataset. **(B)** Gene set enrichment analysis (GSEA) of DEGs showed the relationship between TCF7L2 and mTORC1 signaling pathway. **(C)** The correlation between TCF7L2 and key genes of AKT/mTOR pathway in TCGA. **(D)** The dysregulation of AKT/mTOR pathway at the protein level in TCF knockdown and overexpression cell lines. E and **F.** AKT inhibitor MK-2206 and mTOR inhibitor Rapamycin were used for rescue experiments in SK1 (**E**) and huh7 (**F**) cells (*n* = 3 biological replicates). *** *P* < 0.001

To elucidate the mechanism of TCF7L2 regulation in HCC, we analyzed the differentially expressed genes (DEGs) between the TCF7L2 knockdown group and the control group in the GSE46465 dataset (Fig. 4A). Further Gene set enrichment analysis (GSEA) of DEGs revealed that TCF7L2 could upregulate the mTORC1 signaling pathway (Fig. 4B). AKT is directly upstream of the mTORC1 signaling pathway, and the AKT/mTOR pathway is activated in a variety of tumors(Popova and Jücker 2021). Therefore, we further investigated whether TCF7L2 can activate the AKT/mTOR pathway in HCC. Pearson correlation was used to analyze the correlation between TCF7L2 and key genes of AKT/mTOR pathway in TCGA, and we found that TCF7L2 was associated with AKT1 and MTOR (Fig. 4C). We further detected AKT/mTOR pathway activation in TCF7L2 knockdown and overexpression cell lines. The results showed that TCF7L2 knockdown decreased the phosphorylation levels of AKT and mTOR, while the phosphorylation levels of AKT and mTOR increased after TCF7L2 overexpression (Fig. 4D). These results suggest that TCF7L2 activates the AKT/mTOR pathway in HCC. To determine whether TCF7L2 regulates migration and invasion via AKT/mTOR pathway in HCC, SK1 and Huh7 cells overexpressing TCF7L2 were treated with the AKT inhibitor MK-2206 and the mTOR inhibitor Rapamycin. We found that both MK-2206 and Rapamycin could significantly inhibit the migration and invasion ability in TCF7L2 overexpressed cell lines (Fig. 4E, F). These results suggest that TCF7L2 promotes HCC migration and invasion by activating AKT/mTOR pathway.

### TCF7L2 regulates transcription of NEDD9

**Fig. 5:**
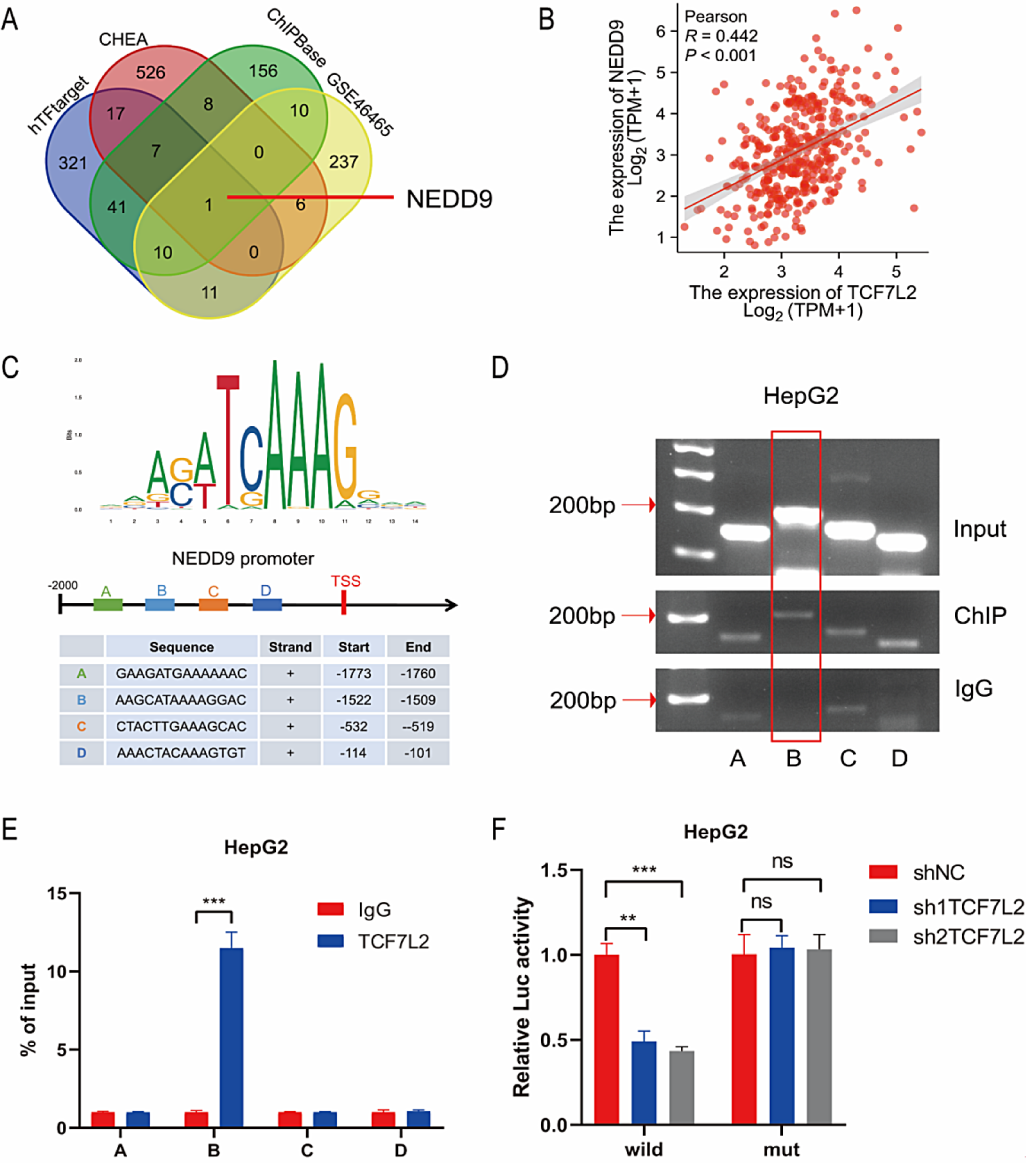
TCF7L2 regulates transcription of NEDD9. **A**. Target gene of TCF7L2 were predicted by three transcription factor databases (hTFtarget, CHEA and ChIPBase) and GSE46465. B. The correlation between the expression of TCF7L2 and NEDD9 in HCC was analyzed in TCGA database. **C**. The binding sites of TCF7L2 and NEDD9 gene promoter from JASPAR database. **D** and **E**. The binding sites of TCF7L2 and NEDD9 gene promoter were analyzed by agarose gel electrophoresis (**D**) and ChIP-qPCR (**E**) in HepG2 cells (*n* = 3 biological replicates). **F**. The fluorescence activity of NEDD9 promoter was analyzed by luciferase report assay in HepG2 cells (*n* = 3 biological replicates). Ns: Not Significant, *P* > 0.05, ** *P* < 0.01, *** *P* < 0.001

Given that TCF7L2 acts as a transcription factor, it mainly plays a role in regulating the transcription of downstream genes. We attempted to find out the directly downstream target genes of TCF7L2. The target genes of TCF7L2 were predicted by three transcription factor databases (hTFtarget, CHEA and ChIPBase), and intersected with the DEGs obtained from GSE46465, we finally identified the TCF7L2 target gene NEDD9 (Fig. 5A). The expression data in the TCGA database also showed that there is a significant correlation between the expression of TCF7L2 and NEDD9 in HCC (Fig. 5B). In addition, the protein level of NEDD9 was altered after TCF7L2 knockdown or overexpression (Figure S2D). Next, we predicted the binding site of TCF7L2 to NEDD9 gene promoter in the JASPAR database (Castro-Mondragon et al. 2022). Four TCF7L2 binding sites with the highest score on the NEDD9 promoter was selected for subsequent analysis: “GAAGATGAAAAAAC” (site A), “AAGCATAAAAGGAC” (site B), “CTACTTGAAAGCAC” (site C) and “AAACTACAAAGTGT” (site D) (Fig. 5C). We designed primers at both ends of each site and analyzed the abundance of each site by agarose gel electrophoresis and ChIP-qPCR. The results showed that compared with IgG group, the abundance of site B was significantly increased (Fig. 5D, E). Meanwhile, luciferase report assays showed that TCF7L2 knockdown significantly inhibited the activity of luciferase carrying site B, while the activity of luciferase carrying site B mutation was not affected by TCF7L2 expression (Fig. 5F). These results indicate that TCF7L2 can directly bind to the promoter region of NEDD9 and positively regulate the transcription of NEDD9.

### TCF7L2 promotes HCC migration and invasion via activating AKT/mTOR pathway through NEDD9

**Fig. 6:**
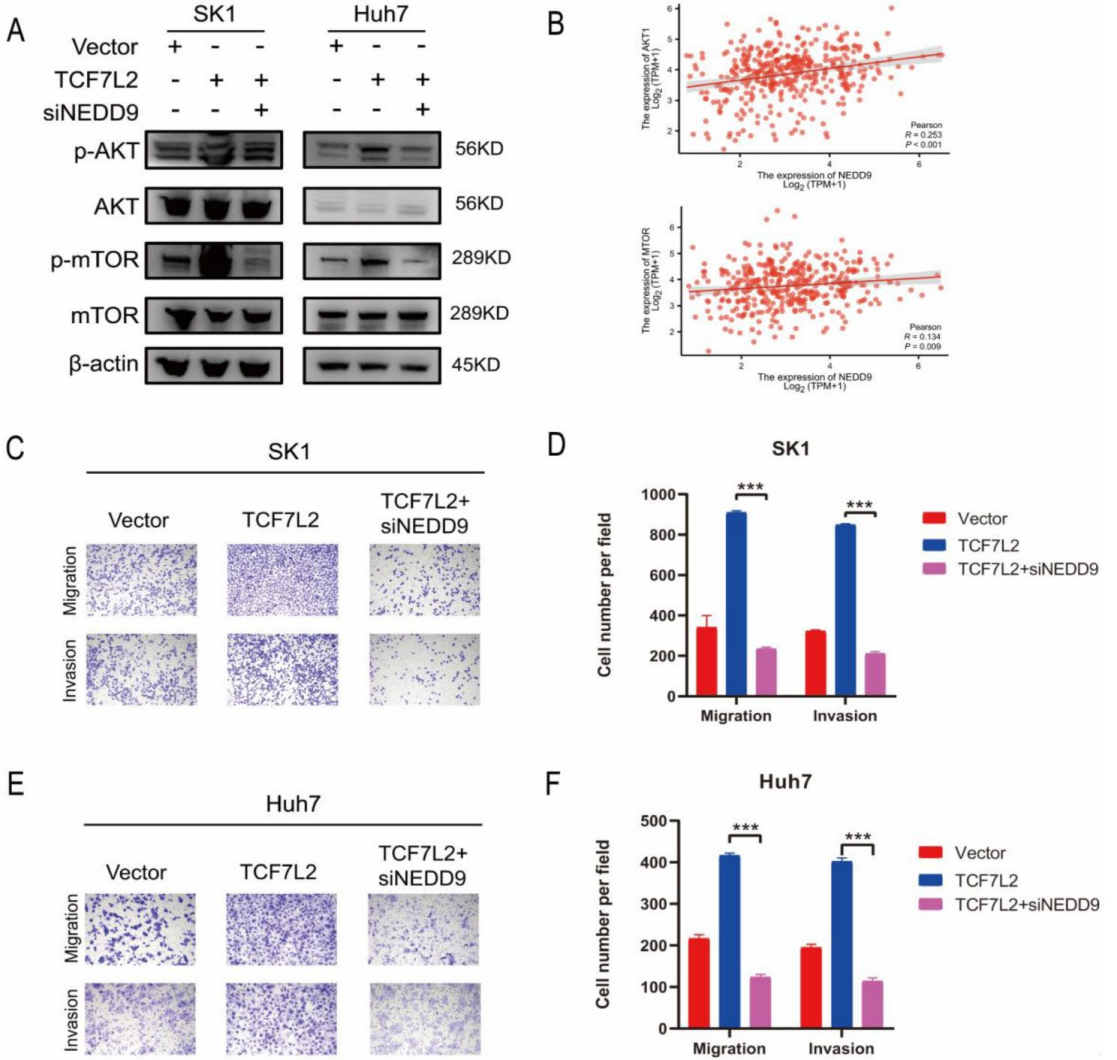
TCF7L2 promotes HCC migration and invasion via activating AKT/mTOR pathway through NEDD9. **A**. Western blotting was performed in SK1 and Huh7 cells to detect the effect of the addition of siNEDD9 on the AKT/mTOR pathway;. **B**. The correlation between the expression of NEDD9 and AKT1 (above) and MTOR (below) in HCC was analyzed in TCGA database. **C** and **D**. Transwell migration and invasion assays were performed on TCF7L2 overexpressed SK1 cells with or without siNEDD9 (*n* = 3 biological replicates). **E** and **F**. Transwell migration and invasion assays were performed on TCF7L2 overexpressed Huh7 cells with or without siNEDD9 (*n* = 3 biological replicates). *** *P* < 0.001

Previous studies have found that NEDD9 can activate the AKT/mTOR pathway and affect tumor progression, but no relevant studies have been reported in HCC (Izumchenko et al. 2009; Xue et al. 2020). To further clarify the function of NEDD9 and its relationship with AKT/mTOR pathway, we constructed NEDD9 small interfering RNA and selected the best interfering effect for subsequent experiments. Rescue experiments were performed in SK1 and Huh7 cell lines with TCF7L2 overexpression, and we found that NEDD9 knockdown significantly inhibited the activation of AKT/mTOR pathway by TCF7L2 overexpression (Fig. 6A). Meanwhile, analysis from the TCGA database also showed the positive correlation of NEDD9 with AKT1 and MTOR (Fig. 6B). To further clarify whether NEDD9 is involved in the pro-metastasis effect of TCF7L2, transwell migration and invasion assays were performed on TCF7L2 overexpressed cells with or without interference of NEDD9. The results showed that NEDD9 interference significantly reduced the migration and invasion ability of SK1 and Huh7 cells after overexpression of TCF7L2 (Fig. 6C-F). These evidences above indicate that TCF7L2 promotes HCC migration and invasion in a NEDD9-dependent manner.

### NEDD9 is associated with TCF7L2 in HCC

**Fig. 7:**
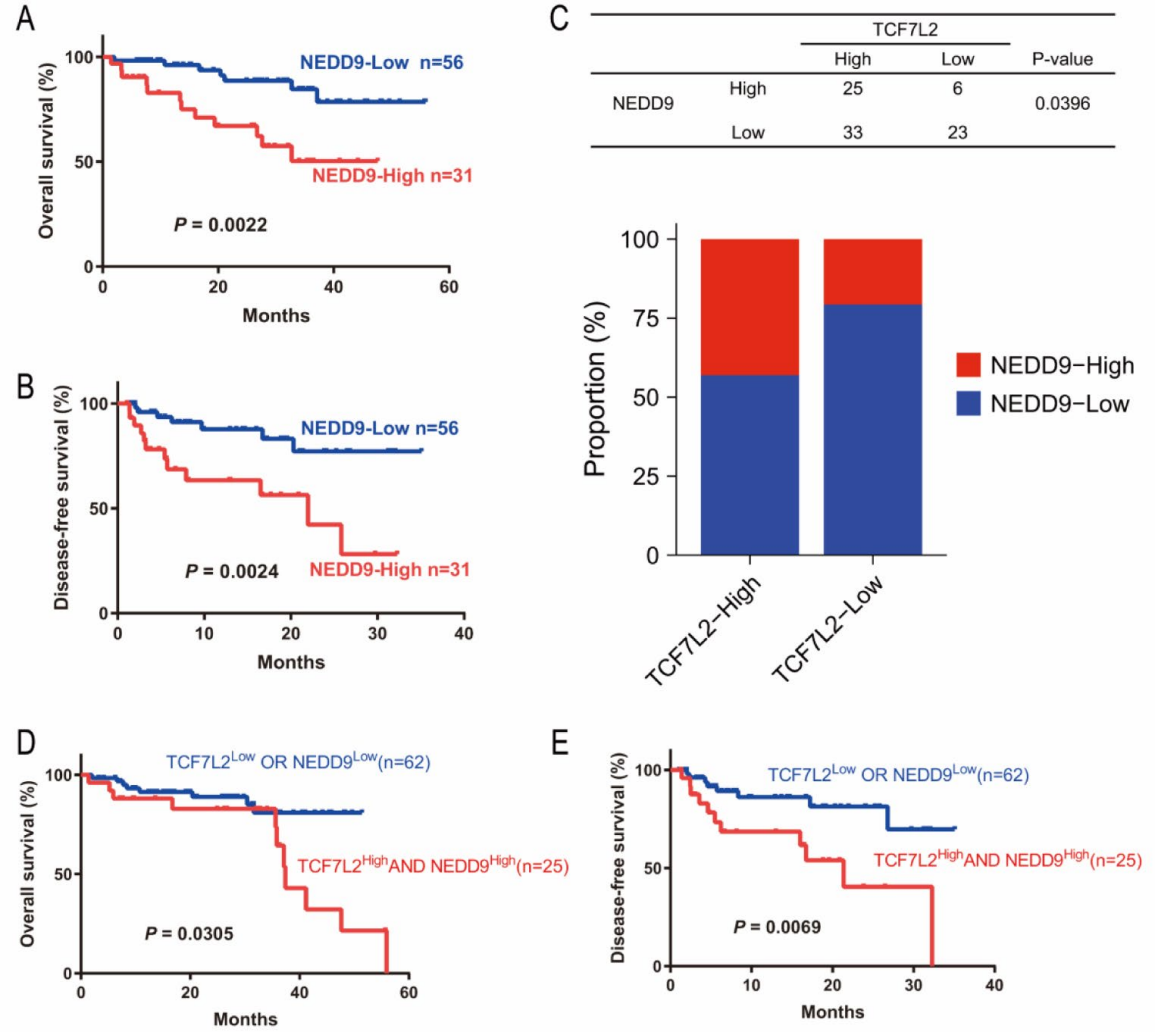
Correlation of NEDD9 and TCF7L2 in HCC. **A** and **B.** Kaplan-Meier survival analysis was performed on overall survival (**A**) and disease-free survival (**B**) of HCC patients with high NEDD9 expression and low NEDD9 expression. **C.** The correlation between the expression of NEDD9 and TCF7L2 in 87 HCC patients. χ2 test were used for assessment of correlation. **D** and **E.** Kaplan-Meier survival analysis was performed on overall survival (**D**) and disease-free survival (**E**) of HCC patients with high expression of both TCF7L2 and NEDD9 (*n* = 25) and those with low expression of at least one of them

We further performed expression and survival analysis in 87 patients with tissue microarray. The results suggested that HCC patients with high NEDD9 expression had significantly higher overall and disease-free survival (Fig. 7A, B). We further analyzed the correlation between NEDD9 and TCF7L2 in 87 HCC patients, and found that NEDD9 was positively correlated with TCF7L2 (Fig. 7C). Furthermore, we investigated the predictive ability of the combination of the two genes on the prognosis of HCC patients. 87 HCC patients were divided into two groups: those with high expression of both TCF7L2 and NEDD9 (*n* = 25) and those with low expression of at least one of them (*n* = 62). The overall and disease-free survival were significantly lower in the group with high expression of both genes than in the group with low expression of at least one gene (Fig. 7D, E), indicating that the combined detection of TCF7L2 and NEDD9 can well predict the prognosis of HCC patients.

## Discussion

As one of the most common malignant tumors, HCC has a very high mortality rate. At present, the treatment of HCC gradually expanded, but the prognosis of patients has not been significantly improved (Couri and Pillai 2019). Therefore, elucidating the mechanism of the progression of HCC and seeking new therapeutic targets for HCC are still the focus of current research. TCF7L2 has been associated with glucose metabolism in the past, especially in type 2 diabetes (Bosque-Plata et al. 2022). However, its role in the development of HCC remains unclear. In present study, TCF7L2 is found to be upregulated in HCC, portending a worse prognosis for these patients. Mechanically, TCF7L2 activates NEDD9 transcription by directly binding to NEDD9 promoter region, and NEDD9 can activate the AKT/mTOR pathway, thereby promoting the migration and invasion of HCC.

TCF7L2 plays a vital role in cell proliferation and stemness maintenance (Hrckulak et al. 2018; Es et al. 2012). Abnormal expression of TCF7L2 has been revealed in kinds of tumors. In colorectal cancer, the absence of TCF7L2 could promote tumor metastasis(Wenzel et al. 2020). Jing et al. found that TCF7L2 was overexpressed in glioma and correlated with poor survival (Jing et al. 2021). In gastric cancer, the expression of TCF7L2 was upregulated, promoting the progression of gastric cancer by forming a positive feedback loop with Wnt7B (Gao et al. 2021). These studies suggest the important role of TCF7L2 in tumor. In line with these, our study uncovered that the expression of TCF7L2 was significantly upregulated in HCC and indicated a poor prognosis. Previous study by Jiang et al. showed that TCF7L2 was highly expressed in HCC, mainly in metastatic tumors (Castro-Mondragon et al. 2022). This finding is also consistent with our research, which found that TCF7L2 promotes HCC metastasis in vivo and in vitro. Therefore, the current study suggests the possibility of TCF7L2 as a biomarker for HCC, especially metastatic HCC.

The AKT/mTOR signaling pathway is involved in regulating many physiological processes in cells, including cell proliferation, growth, metabolism, and movement. Studies have found that activated AKT can phosphorylate mTORC1 at Ser2448 and thus activate mTORC1, which is essential for cell proliferation(Wendel et al. 2004). In fact, the activation of AKT/mTOR pathway has been found in a variety of tumors, and the activation of AKT/mTOR pathway is present in more than half of HCC patients (Wang et al. 2023). In present study, we found that TCF7L2 upregulated cells were significantly enriched in the mTORC1 pathway by GSEA analysis. We demonstrated that upregulation of TCF7L2 activated the AKT/mTOR pathway at the protein level, while AKT/mTOR pathway was inhibited after TCF7L2 knockdown. Functionally, we found that the pro-metastatic effect of TCF7L2 on HCC could be reversed by AKT or mTOR inhibitors, indicating that TCF7L2 plays a role in HCC by activating the AKT/mTOR pathway. Our findings are consistent with those of Wu et al., who found that TCF7L2 could activate the AKT/mTOR pathway in breast cancer (Wu et al. 2021). Our study suggests the crosstalk between the Wnt pathway and the AKT/mTOR pathway in HCC, and the combined targeting of these two pathways for the treatment of HCC deserves further investigation.

NEDD9 is a scaffold protein involved in cell signal transduction, which can regulate a variety of cellular processes, including cell growth, proliferation, injury and metastasis (Shagisultanova et al. 2015; Tikhomirova et al. 2022). A series of previous studies have found that NEDD9 can affect epithelial mesenchymal transformation and promote tumor metastasis (Li et al. 2016; Gabbasov et al. 2018; Deneka et al. 2022; Wang et al. 2019). Zhou et al. found that NEDD9 is upregulated in HCC, and the high expression of NEDD9 could promote the metastasis of HCC (Zhou et al. 2017a, b). In our clinical sample, patients with high NEDD9 expression had significantly worse overall survival and disease-free survival than patients with low NEDD9 expression. The expression of NEDD9 is regulated by upstream genes. In melanoma, TCF7L2 regulates the expression of NEDD9 through miRNA to affect tumor progression (Rambow et al. 2016). Our study found that NEDD9 is a direct target gene of TCF7L2, and TCF7L2 can directly bind to the -1522/-1509 site of the NEDD9 promoter and positively regulate the transcription of NEDD9. Downstream of NEDD9 is often associated with the activation of several kinases, including FAK, Src, and AURKA (Izumchenko et al. 2009; Sima et al. 2013; Pugacheva et al. 2007). Deneka et al. found that NEDD9 was involved in the activation of AKT in lung cancer (Deneka et al. 2021). Our results suggest that TCF7L2 activates AKT/mTOR pathway by regulating NEDD9, thereby promoting HCC metastasis. However, how NEDD9 activates the AKT/mTOR pathway in HCC remains unclear which deserves further exploration.

## Conclusions

In summary, this study elucidates the role and mechanism of TCF7L2 in HCC metastasis. TCF7L2 targets NEDD9 transcription to activate AKT/mTOR pathway, thereby promoting HCC migration and invasion. Our findings elucidate the mechanism of action of TCF7L2 in HCC, suggesting the potential of TCF7L2 as a prognostic marker and therapeutic target for HCC.

### Electronic supplementary material

Below is the link to the electronic supplementary material.

Supplementary Material 1

Supplementary Material 2

Supplementary Material 3

Supplementary Material 4

## Data Availability

Data from the current study are all available.

## Abbreviations

TCF7L2: Transcription factor 7 like 2
HCC: Hepatocellular Carcinoma
NEDD9: Neural precursor cell Expressed Developmentally Downregulated protein 9
TCF/LEF: T cell factor/Lymphoid Enhancer Factor
GEO: Gene Expression Omnibus
TCGA: The Cancer Genome Atlas
FBS: Fetal Bovine Serum
SDS-PAGE: Sodium Dodecyl Sulfate-Polyacrylamide Gel Electrophoresis
shRNA: Small hairpin RNA
siRNA: Small interfering RNA
CCK8: Cell Counting kit-8
ChIP: Chromatin Immunoprecipitation
GSEA: Gene Set Enrichment Analysis

## References

[CR6] Anastas JN, Moon RT. WNT signalling pathways as therapeutic targets in cancer. Nat Rev Cancer. 2013;13:11–26. 10.1038/nrc3419. 23258168 10.1038/nrc3419

[CR25] Castro-Mondragon JA, Riudavets-Puig R, Rauluseviciute I et al. (2022) JASPAR 2022: The 9th release of the open-access database of transcription factor binding profiles. Nucleic Acids Res 50:D165–D173. 10.1093/nar/gkab1113. 10.1093/nar/gkab1113 PMC8728201 34850907

[CR21] Chen J, Ding C, Chen Y, et al. ACSL4 reprograms fatty acid metabolism in hepatocellular carcinoma via c-Myc/SREBP1 pathway. Cancer Lett. 2021;502:154–65. 10.1016/j.canlet.2020.12.019. 33340617 10.1016/j.canlet.2020.12.019

[CR28] Couri T, Pillai A. Goals and targets for personalized therapy for HCC. Hepatol Int. 2019;13:125–37. 10.1007/s12072-018-9919-1. 30600478 10.1007/s12072-018-9919-1

[CR29] del Bosque-Plata L, Hernández-Cortés EP, Gragnoli C. The broad pathogenetic role of TCF7L2 in human diseases beyond type 2 diabetes. J Cell Physiol. 2022;237:301–12. 10.1002/jcp.30581. 34612510 10.1002/jcp.30581 PMC9292842

[CR47] Deneka AY, Kopp MC, Nikonova AS, et al. Nedd9 restrains autophagy to Limit Growth of Early Stage non–small cell Lung Cancer. Cancer Res. 2021;81:3717–26. 10.1158/0008-5472.CAN-20-3626. 34006524 10.1158/0008-5472.CAN-20-3626 PMC8277748

[CR41] Deneka AY, Nikonova AS, Lee HO, et al. NEDD9 sustains hexokinase expression to promote glycolysis. Oncogenesis. 2022;11:1–8. 10.1038/s41389-022-00391-w. 35410460 10.1038/s41389-022-00391-w PMC9001639

[CR14] Drake I, Wallström P, Hindy G, et al. TCF7L2 type 2 diabetes risk variant, lifestyle factors, and incidence of prostate cancer. Prostate. 2014;74:1161–70. 10.1002/pros.22832. 24961829 10.1002/pros.22832

[CR40] Gabbasov R, Xiao F, Howe CG, et al. NEDD9 promotes oncogenic signaling, a stem/mesenchymal gene signature, and aggressive ovarian cancer growth in mice. Oncogene. 2018;37:4854–70. 10.1038/s41388-018-0296-y. 29773902 10.1038/s41388-018-0296-y PMC6119087

[CR33] Gao Q, Yang L, Shen A, et al. A WNT7B-m6A-TCF7L2 positive feedback loop promotes gastric cancer progression and metastasis. Signal Transduct Target Ther. 2021;6:2020–2. 10.1038/s41392-020-00397-z. 10.1038/s41392-020-00397-z PMC7851143 33526767

[CR7] Hrckulak D, Kolar M, Strnad H, Korinek V. (2016) TCF/LEF transcription factors: an update from the internet resources. Cancers (Basel). 8. 10.3390/cancers8070070 PMC4963812 27447672

[CR30] Hrckulak D, Janeckova L, Lanikova L, et al. Wnt effector TCF4 is dispensable for wnt signaling in human cancer cells. Genes (Basel). 2018;9. 10.3390/genes9090439. 10.3390/genes9090439 PMC6162433 30200414

[CR26] Izumchenko E, Singh MK, Plotnikova OV, et al. NEDD9 promotes Oncogenic Signaling in Mammary Tumor Development. Cancer Res. 2009;69:7198–206. 10.1158/0008-5472.CAN-09-0795. 19738060 10.1158/0008-5472.CAN-09-0795 PMC2758619

[CR8] Jin T. Current understanding on role of the wnt signaling pathway effector TCF7L2 in glucose homeostasis. Endocr Rev. 2016;37:254–77. 10.1210/er.2015-1146. 27159876 10.1210/er.2015-1146

[CR32] Jing S, Chen L, Han S, et al. Expression of TCF7L2 in Glioma and its relationship with clinicopathological characteristics and patient overall survival. Front Neurol. 2021;12:1–7. 10.3389/fneur.2021.627431. 10.3389/fneur.2021.627431 PMC8296806 34305772

[CR17] Lachmann A, Xu H, Krishnan J, et al. ChEA: transcription factor regulation inferred from integrating genome-wide ChIP-X experiments. Bioinformatics. 2010;26:2438–44. 10.1093/bioinformatics/btq466. 20709693 10.1093/bioinformatics/btq466 PMC2944209

[CR10] Lee DS, An TH, Kim H, et al. Tcf7l2 in hepatocytes regulates de novo lipogenesis in diet-induced non-alcoholic fatty liver disease in mice. Diabetologia. 2023;931–54. 10.1007/s00125-023-05878-8. 10.1007/s00125-023-05878-8 PMC10036287 36759348

[CR39] Li P, Sun T, Yuan Q, et al. The expressions of NEDD9 and E-cadherin correlate with metastasis and poor prognosis in triple-negative breast cancer patients. Onco Targets Ther. 2016;9:5751–9. 10.2147/OTT.S113768. 27703373 10.2147/OTT.S113768 PMC5036611

[CR23] Li T, Fu J, Zeng Z, et al. TIMER2.0 for analysis of tumor-infiltrating immune cells. Nucleic Acids Res. 2020;48:W509–14. 10.1093/NAR/GKAA407. 32442275 10.1093/NAR/GKAA407 PMC7319575

[CR15] Malakar P, Stein I, Saragovi A, et al. Long noncoding RNA MALAT1 regulates cancer glucose metabolism by enhancing mTOR-Mediated translation of TCF7L2. Cancer Res. 2019;79:2480–93. 10.1158/0008-5472.CAN-18-1432. 30914432 10.1158/0008-5472.CAN-18-1432

[CR24] Popova NV, Jücker M. The role of mtor signaling as a therapeutic target in cancer. Int J Mol Sci. 2021;22:1–30. 10.3390/ijms22041743. 10.3390/ijms22041743 PMC7916160 33572326

[CR46] Pugacheva EN, Jablonski SA, Hartman TR, et al. HEF1-Dependent Aurora a activation induces disassembly of the primary cilium. Cell. 2007;129:1351–63. 10.1016/j.cell.2007.04.035. 17604723 10.1016/j.cell.2007.04.035 PMC2504417

[CR44] Rambow F, Bechadergue A, Luciani F, et al. Regulation of Melanoma Progression through the TCF4/miR-125b/NEDD9 Cascade. J Invest Dermatol. 2016;136:1229–37. 10.1016/j.jid.2016.02.803. 26968260 10.1016/j.jid.2016.02.803

[CR18] Rouillard AD, Gundersen GW, Fernandez NF et al. (2016) The harmonizome: a collection of processed datasets gathered to serve and mine knowledge about genes and proteins. Database (Oxford) 2016:1–16. 10.1093/database/baw100. 10.1093/database/baw100 PMC4930834 27374120

[CR37] Shagisultanova E, Gaponova AV, Gabbasov R, et al. Preclinical and clinical studies of the NEDD9 scaffold protein in cancer and other diseases. Gene. 2015;567:1–11. 10.1016/j.gene.2015.04.086. 25967390 10.1016/j.gene.2015.04.086 PMC4458429

[CR45] Sima N, Cheng X, Ye F, et al. The overexpression of scaffolding protein NEDD9 promotes Migration and Invasion in Cervical Cancer via Tyrosine Phosphorylated FAK and SRC. PLoS ONE. 2013;8:1–12. 10.1371/journal.pone.0074594. 10.1371/journal.pone.0074594 PMC3776827 24058594

[CR1] Sung H, Ferlay J, Siegel RL, et al. Global Cancer statistics 2020: GLOBOCAN estimates of incidence and Mortality Worldwide for 36 cancers in 185 countries. CA Cancer J Clin. 2021;71:209–49. 10.3322/caac.21660. 33538338 10.3322/caac.21660

[CR3] Tang L, Chen R, Xu X. Synthetic lethality: a promising therapeutic strategy for hepatocellular carcinoma. Cancer Lett. 2020;476:120–8. 10.1016/j.canlet.2020.02.016. 32070778 10.1016/j.canlet.2020.02.016

[CR2] Tang L, Wei R, Chen R, et al. Establishment and validation of a cholesterol metabolism-related prognostic signature for hepatocellular carcinoma. Comput Struct Biotechnol J. 2022;20:4402–14. 10.1016/j.csbj.2022.07.030. 36051877 10.1016/j.csbj.2022.07.030 PMC9420502

[CR38] Tikhomirova M, Topchu I, Mazitova A, et al. NEDD9 restrains dsDNA damage response during Non-small Cell Lung Cancer (NSCLC) Progression. Cancers (Basel). 2022;14. 10.3390/cancers14102517. 10.3390/cancers14102517 PMC9139181 35626121

[CR31] van Es JH, Haegebarth A, Kujala P, et al. A critical role for the wnt Effector Tcf4 in adult Intestinal Homeostatic Self-Renewal. Mol Cell Biol. 2012;32:1918–27. 10.1128/mcb.06288-11. 22393260 10.1128/mcb.06288-11 PMC3347420

[CR5] Vilchez V, Turcios L, Marti F, Gedaly R. Targeting Wnt/β-catenin pathway in hepatocellular carcinoma treatment. World J Gastroenterol. 2016;22:823–32. 10.3748/wjg.v22.i2.823. 26811628 10.3748/wjg.v22.i2.823 PMC4716080

[CR42] Wang Y, Bibi M, Min P, et al. SOX2 promotes hypoxia-induced breast cancer cell migration by inducing NEDD9 expression and subsequent activation of Rac1/HIF-1α signaling. Cell Mol Biol Lett. 2019;24:1–12. 10.1186/s11658-019-0180-y. 31462898 10.1186/s11658-019-0180-y PMC6704701

[CR13] Wang Y, Men X, Gu Y, et al. Haplotype analysis on correlation between transcription factor 7-like 2 gene polymorphism and breast cancer risk. BMC Cancer. 2021;21:1–6. 10.1186/s12885-021-08571-4. 34340678 10.1186/s12885-021-08571-4 PMC8327437

[CR35] Wang C, Cao F, Cao J, et al. CD58 acts as a tumor promotor in hepatocellular carcinoma via activating the AKT/GSK-3β/β-catenin pathway. J Transl Med. 2023;21:539. 10.1186/s12967-023-04364-4. 37573318 10.1186/s12967-023-04364-4 PMC10422835

[CR34] Wendel HG, De Stanchina E, Fridman JS, et al. Survival signalling by Akt and eIF4E in oncogenesis and cancer therapy. Nature. 2004;428:332–7. 10.1038/nature02369. 15029198 10.1038/nature02369

[CR11] Wenzel J, Rose K, Haghighi EB, et al. Loss of the nuclear wnt pathway effector TCF7L2 promotes migration and invasion of human colorectal cancer cells. Oncogene. 2020;39:3893–909. 10.1038/s41388-020-1259-7. 32203164 10.1038/s41388-020-1259-7 PMC7203011

[CR36] Wu D, Jia H, Zhang Z, Li S. Circ-PRMT5 promotes breast cancer by the miR-509-3p/TCF7L2 axis activating the PI3K/AKT pathway. J Gene Med. 2021;23:1–12. 10.1002/jgm.3300. 10.1002/jgm.3300 33277756

[CR27] Xue Y, Zhong Y, Wu T, et al. Anti-proliferative and apoptosis-promoting effect of microrna-125b on pancreatic cancer by targeting nedd9 via pi3k/akt signaling. Cancer Manag Res. 2020;12:7363–73. 10.2147/CMAR.S227315. 32903925 10.2147/CMAR.S227315 PMC7445537

[CR16] Zhang Q, Liu W, Zhang HM, et al. hTFtarget: a comprehensive database for regulations of human transcription factors and their targets. Genomics Proteom Bioinforma. 2020;18:120–8. 10.1016/j.gpb.2019.09.006. 10.1016/j.gpb.2019.09.006 PMC7647694 32858223

[CR9] Zhang Z, Xu L, Xu X. The role of transcription factor 7-like 2 in metabolic disorders. Obes Rev. 2021;22:1–15. 10.1111/obr.13166. 10.1111/obr.13166 33615650

[CR4] Zhang QJ, Li DZ, Lin BY, et al. SNHG16 promotes hepatocellular carcinoma development via activating ECM receptor interaction pathway. Hepatobiliary Pancreat Dis Int. 2022a;21:41–9. 10.1016/j.hbpd.2021.09.006. 34600815 10.1016/j.hbpd.2021.09.006

[CR12] Zhang T, Wang B, Su F, et al. TCF7L2 promotes anoikis resistance and metastasis of gastric cancer by transcriptionally activating PLAUR. Int J Biol Sci. 2022b;18:4560–77. 10.7150/ijbs.69933. 35864968 10.7150/ijbs.69933 PMC9295057

[CR19] Zhou KR, Liu S, Sun WJ, et al. ChIPBase v2.0: decoding transcriptional regulatory networks of non-coding RNAs and protein-coding genes from ChIP-seq data. Nucleic Acids Res. 2017a;45:D43–50. 10.1093/nar/gkw965. 27924033 10.1093/nar/gkw965 PMC5210649

[CR43] Zhou S, Xu M, Shen J, et al. Overexpression of NEDD9 promotes cell invasion and metastasis in hepatocellular carcinoma. Clin Res Hepatol Gastroenterol. 2017b;41:677–86. 10.1016/j.clinre.2017.04.011. 28578938 10.1016/j.clinre.2017.04.011

[CR20] Zhuo J, Lu D, Lin Z, et al. The distinct responsiveness of cytokeratin 19-positive hepatocellular carcinoma to regorafenib. Cell Death Dis. 2021;12. 10.1038/s41419-021-04320-4. 10.1038/s41419-021-04320-4 PMC8595883 34785656

[CR22] Zuo Q, He J, Zhang S, et al. PPARγ Coactivator-1α suppresses metastasis of Hepatocellular Carcinoma by Inhibiting Warburg Effect by PPARγ–Dependent WNT/β-Catenin/Pyruvate dehydrogenase kinase isozyme 1 Axis. Hepatology. 2021;73:644–60. 10.1002/hep.31280. 32298475 10.1002/hep.31280

