# Supplementary material for: Transcription factor 7 like 2 promotes metastasis in hepatocellular carcinoma via NEDD9-mediated activation of AKT/mTOR signaling pathway
Source: Mol Med. 2024 Jul 25;30:108. doi: 10.1186/s10020-024-00878-9 (PMC11282612; doi:10.1186/s10020-024-00878-9)
Supplement: Supplementary file 1 — Supplementary Material 1 [file 10020_2024_878_MOESM1_ESM.docx]

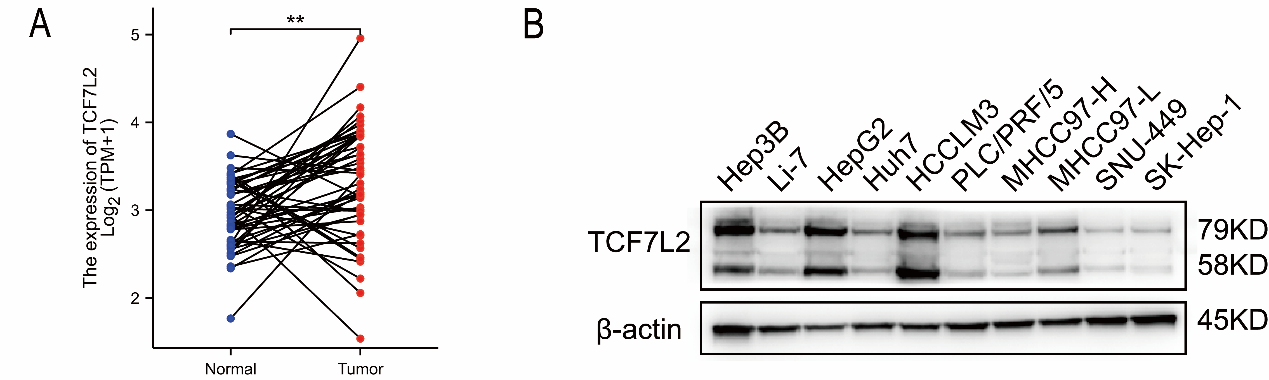


**Figure S1. The mRNA expression level of TCF7L2 in HCC.**

**A.** The mRNA expression level of TCF7L2 in tumors tissues and adjacent normal liver tissues from TCGA database.

**B.** The protein expression level of TCF7L2 in different HCC cell lines. ** *P* < 0.01.
