# Supplementary material for: Transcription factor 7 like 2 promotes metastasis in hepatocellular carcinoma via NEDD9-mediated activation of AKT/mTOR signaling pathway
Source: Mol Med. 2024 Jul 25;30:108. doi: 10.1186/s10020-024-00878-9 (PMC11282612; doi:10.1186/s10020-024-00878-9)
Supplement: Supplementary file 2 — Supplementary Material 2 [file 10020_2024_878_MOESM2_ESM.docx]

**Supplementary Table S1. Clinical characteristics of 87 HCC patients.**

| **Clinical characteristics** | **Information** |
| --- | --- |
| Gender | Male : Female = 80 : 7 |
| Age (years，mean ± SD) | 54.6 ± 11.8 |
| Tumor size (cm) | (> 5) : (≦ 5) = 40 : 47 |
| Tumor number | Single : Multiple = 67 : 20 |
| Cirrhosis | Yes : No = 45 : 42 |
| HBsAg | Positive : Negative = 78 : 9 |
| Vascular invasion | Yes : No = 48 : 39 |
| AFP (ng/mL) | (> 400) : (≦ 400) = 39 : 48 |
| TNM stage | I&II : III&IV = 52 : 35 |
