# Supplementary material for: Transcription factor 7 like 2 promotes metastasis in hepatocellular carcinoma via NEDD9-mediated activation of AKT/mTOR signaling pathway
Source: Mol Med. 2024 Jul 25;30:108. doi: 10.1186/s10020-024-00878-9 (PMC11282612; doi:10.1186/s10020-024-00878-9)
Supplement: Supplementary file 3 — Supplementary Material 3 [file 10020_2024_878_MOESM3_ESM.doc]

**Supplementary Table S2. Sequences of primers and siRNAs and shRNAs.**

| **1. Primers used in qRT-PCR and ChIP-qPCR analysis** | | |  |  |
| --- | --- | --- | --- | --- |
| **Gene** | **Forward Primer** | **Reverse Primer** |  |  |
| β-actin | CACCATTGGCAATGAGCGGTTC | AGGTCTTTGCGGATGTCCACGT |  |  |
| TCF7L2 | GACAAGCAGCCGGGAGAGACCAATG | GGGGGAGGCGAATCTAGTAAGCT |  |  |
| NEDD9 | GACCGTCATAGAGCAGAACAC | TGCATGGGACCAATCAGAAGC |  |  |
| NEDD9-A | GAAGTGGGAAATGAAGCCATAGGA | GCCATCAAATTGTCTGCATAAGTC |  |  |
| NEDD9-B | GACTTATGCAGACAATTTGATGGC | TGGAGGTAGAGAAGTCTGGATAGT |  |  |
| NEDD9-C | AATTCAAACCCAGGAGGCTTGTCT | ATGCTAGAGGCTAGGTGCTCAGAG |  |  |
| NEDD9-D | AAGAGGCCAGATGAGAACAAGCT | GGTTGCTGTGAGGTGATGTTGTC |  |  |
| **2. siRNAs** |  |  |  |  |
| **Gene** | **Forward** | **Reverse** |  |  |
| si-NEDD9-1 | GGGCCUUAUAUGACAAUGUTT TT | ACAUUGUCAUAUAAGGCCCTT |  |  |
| si-NEDD9-2 | CCAGGACAUUCGCAACAAATT | UUUGUUGCGAAUGUCCUGGTT |  |  |
| si-NEDD9-3 | GGAAAGGGAUGGUGUUUAUTT | UAAACACCAUCCCUUUCCTT |  |  |
| **3. shRNAs** | **Sequence** | |  |  |
| sh1-TCF7L2 | GGGATAACTATGGAAAGAA | |  |  |
| Sh2-TCF7L2 | CACATAAAGAAACCTCTTA | |  |  |
|  | | |  |  |
