# Supplementary material for: Transcription factor 7 like 2 promotes metastasis in hepatocellular carcinoma via NEDD9-mediated activation of AKT/mTOR signaling pathway
Source: Mol Med. 2024 Jul 25;30:108. doi: 10.1186/s10020-024-00878-9 (PMC11282612; doi:10.1186/s10020-024-00878-9)
Supplement: Supplementary file 4 — Supplementary Material 4 [file 10020_2024_878_MOESM4_ESM.docx]

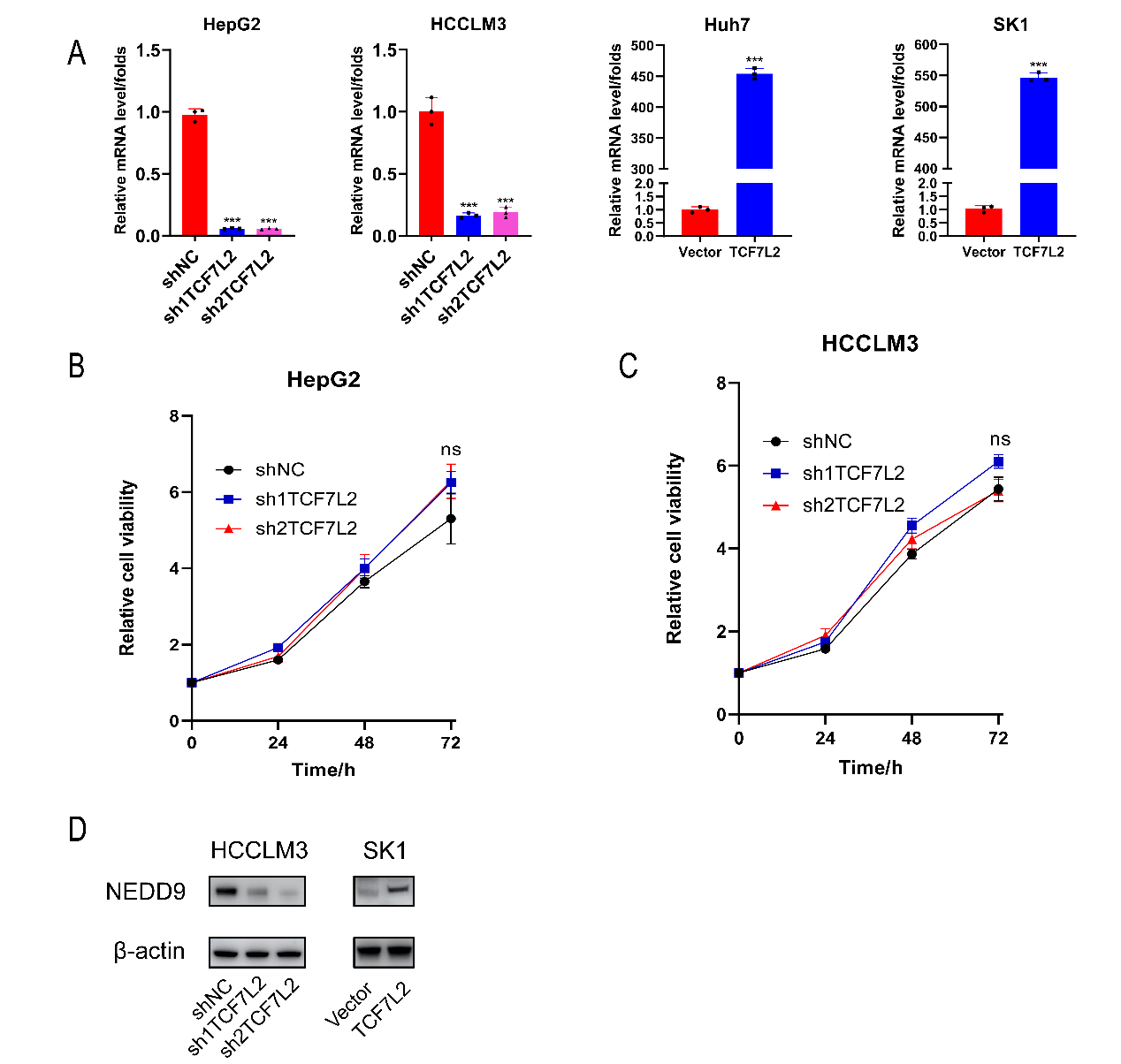


**Figure S2. Knockdown and overexpression of TCF7L2 and its effect on HCC proliferation.**

**A.** The knockdown and overexpression effects of TCF7L2 in HCC cell lines were verified at the mRNA level (n = 3 biological replicates).

**B and C.** CCK8 assays showed that TCF7L2 knockdown did not affect the proliferation capacity of HepG2 (B) and HCCLM3 (C) cell lines (n = 3 biological replicates).

**D.** Changes at protein level of NEDD9 after TCF7L2 knockdown or overexpression by WB assay. Ns: not significant, *P* >0.05, *** *P* < 0.001.
